# Life Affirming Pathways to Aging and Well-Being Among Sexual and Gender Diverse Older Adults

**DOI:** 10.1093/geroni/igaf122.701

**Published:** 2025-12-31

**Authors:** Karen Fredriksen-Goldsen

**Affiliations:** University of Washington, Seattle, Washington, United States

## Abstract

Sexual and gender diverse older adults comprise a growing and resilient, yet health disparate and underserved population. Based on the Health Equity Promotion Model, Dr. Karen Fredriksen- Goldsen, PI, presents findings from the first national, longitudinal 15-year study of this population, Aging with Pride: National Health, Aging and Sexuality/Gender Study (N = 2,450). Utilizing structural equation modeling, the findings identify life affirming pathways to sexual and gender diverse older adults’ well-being and good health. Specific factors, including identity affirmation, social and psychological resources, and health promoting behaviors are associated with positive aging and health outcomes, while the adverse consequences of social exclusion, discrimination and violence are related to poor physical and mental health. The effects of marginalization on identity affirmation are conditional on identity management style, indicating that in case of heightened vulnerabilities (e.g., frailty or poor health) being open about one’s identity may be associated with worse health outcomes, especially in adverse environments. Thus, strategically concealing one’s sexual or gender diverse identity in hostile situations may be socially protective yet may take a toll personally and lead to social isolation. While being consistently open may be psychologically protective, it may increase vulnerability to marginalization and violence. Providers must remain aware of the historical and contemporary contexts and lived experiences of sexual and gender diverse older adults to develop tailored prevention strategies and interventions promoting well-being and good health. The lessons learned from these communities provide important insights for aging well in our increasingly diverse older adult population.

